# Parsing-based approach for entity extraction from natural language software requirements

**DOI:** 10.1371/journal.pone.0361105

**Published:** 2026-10-21

**Authors:** Sabin Amjad, Arooj Saleem, Ahmad Salman Khan, Muhammad Waqas Anwar

**Affiliations:** 1 Department of Software Engineering, The University of Lahore, Lahore, Pakistan; 2 Department of Computer Science, Government College University, Lahore, Pakistan; Infotec: Centro de Investigacion e Innovacion en Tecnologias de la Informacion y Comunicacion, MEXICO

## Abstract

Software requirements are written in natural language, so they are inherently ambiguous. This ambiguity results in the misinterpretation of the requirements and leads to an incorrect implementation of the system that does not meet the user’s needs. So, it is important for all the stakeholders to comprehend the requirements. The requirement statements revolve around the requirement entities. Identifying requirement entities is essential to understand requirements, and they are used in identifying coreferent entities, creating glossaries, producing summaries of documents, feature extraction, requirements classification, and domain understanding. Manual entity extraction is a labour-intensive task. Recently, transformer-based models have played an important role in improving entity extraction results, but they require extensive pre-training of models for domain-specific tasks. To overcome these challenges, the proposed automated approach is used to extract entities from software requirements without the need for manual efforts and a large dataset for pre-training. In this study, two widely used parsing techniques, constituency parsing and dependency parsing, are applied to extract noun phrase-based entities. In addition, a hybrid approach comprising the combined extractions of both parsers is developed. The dataset comprises the software requirements from the healthcare domain and is manually annotated to establish a gold standard for evaluation. Experimental results indicate that the hybrid approach achieves the best overall extraction performance among the evaluated methods. While the constituency parser achieves the highest precision, it exhibits comparatively lower recall. In contrast, the hybrid approach achieves the highest recall and F1-score while maintaining precision comparable to that of the constituency parser. These results demonstrate that the hybrid method provides a more effective solution for automatically extracting entities from software requirements by leveraging syntactic structures of sentences rather than relying on domain-specific model training.

## Introduction

Software requirements cover the scope of the project. Requirements indicate what is to be developed, serving as a guideline for stakeholders and the development team. Software requirements include both the functional and non-functional requirements of the project [1]. Requirements are important for all stakeholders, including technical and business participants. The subsequent software development phases are dependent on the requirements, whether it is the development or operation phase [2]. The requirement engineers write requirements in natural language, which is understandable for all the stakeholders [1 - 3]. Natural language has some inherent properties that lead to quality issues, including inconsistency, incompleteness, and ambiguousness [2, 4].

The needs and requirements refer to the things that are called entities. The terms may belong to different levels, which include business unit, product, system, or system element, and enterprise [5]. Requirement statements have explicit specification of concepts, which are the function objects. The function objects represent the requirements entities in the statements [6]. The process of extracting the core information of the requirement document is called information extraction or entity recognition [7].

In the existing studies, different terms are used to represent the same or similar concept. For the same concept, the terms used are Entity Extraction [1, 6-8], Entity Terms Extraction, and Domain-Specific Entity Terms Extraction [9]. For a similar concept, the terms used are: Requirements Terms Extraction, Terminology extraction [10], Glossary Term Extraction [11, 12], and Requirements-Related Term Extraction [13].

These requirement-specific entities are the noun phrases [6 - 9], whereas glossary terms consist of an entity list and an action list. The entity list consists of concrete nouns, while the action list consists of process nouns and concrete verbs [14]. Some linguistic approaches have extracted only noun phrases as requirement glossary terms [15].

The requirement entities play a key role in the understanding of the requirement concepts. Once the entities are extracted from requirement statements, they are used in information extraction [6 - 8], feature modelling [10], feature extraction [16], size estimation of the project using function point analysis [17], and identifying domain elements for creating various UML diagrams, particularly class diagrams [18]. Requirement entities also play a significant role in improving the quality of software requirements in quality assessment systems [1]. Requirement terms are used to extract domain knowledge for understanding the system domain by the analyst [13]. Glossary term extraction is used in the creation of glossaries [9, 11, 12]. These terms are further used in the identification of term aliases, in which different terms refer to the same concept or entity. These aliases need to be identified to avoid misinterpretation of requirements [4, 9, 12]. These terms should have a common definition in the glossaries to avoid any misunderstandings of technical terms among stakeholders in the project domain [15].

Manual extraction of entities from requirement statements is a laborious and time-consuming task. It also leads to missing and duplicate entity extraction [6, 10]. Heuristics are applied to extract entities from the requirements and find their corresponding match to identify co-referent entities and anaphoric ambiguities. Since the approaches are based on human judgement, they are not suitable for domain-specific tasks [19]. Parsers for Parts of Speech (POS) tagging are trained on generic corpora like public news corpora. There is a need to create a domain-specific dictionary that needs to be integrated into the parser to analyse the words correctly for a domain-specific task [17]. Parser-based approaches are also used in the feature extraction tasks [10]. To extract the requirements-related knowledge based on the terms, a document repository is required [13]. Rule-based approaches are used to identify term aliases [9, 12], extract glossary terms and generate glossaries [11, 15, 20], extract requirements-related knowledge [13], and identify entity terms for domain elements [18]. POS tagging-based techniques have relatively less precision and recall.

A few studies have used Machine Learning (ML) and Deep Learning (DL) based approaches for the Named Entity Recognition (NER) [1, 7], entity extraction [6, 8], and glossary term extraction [21] tasks. The models used are pre-trained on general corpora. As the requirements are domain-specific, the models give the best results for precision and recall evaluation measures when trained on a large volume of domain-specific corpus. Generating a domain-specific corpus requires extensive annotation of large volumes of data. There are chances of inconsistencies and discrepancies in the labelling of data by humans [1, 6, 7].

Different software needs are expressed using various sentence structures, so an automated approach is required to correctly identify the structure of the sentences [18]. The goal of this research is to automatically extract entities from software requirement statements based on sentence structure. An automated syntax-driven approach is proposed to extract software requirement entities by integrating constituency parsing and dependency parsing techniques. The proposed approach does not require additional training on domain-specific data, as it relies on pretrained parsers on general linguistic corpora. It also does not need the extensive configurations of machine learning models and the setting of different parameters. Since the entity extraction approach is based on grammatical structure and does not rely on extensive manual labelling of requirement entities, it is potentially transferable across different domains. An analysis of constituency parsing, dependency parsing, and a hybrid approach is performed to identify the most effective strategy for the entity extraction task.

The following are the contributions of this research:

- Prepare a dataset comprising requirement statements from the healthcare domain to demonstrate the effectiveness of the parsing techniques.
- A list of manually annotated entities serving as the gold standard.
- Using encoder and decoder-based constituency and transition-based dependency parsers to extract noun phrases termed here as requirement entities from software requirements. These parsers are implemented using spaCy and adapted to deal with special characters like hyphens and apostrophes.
- Development and use of a hybrid approach by integrating the results of constituency and dependency parsers after removing duplications to improve the coverage of noun-phrase spans.
- A comparison of the results of the constituency parser, the dependency parser, and the hybrid approach.

The output of the approach is the list of requirement entities from each requirement statement, and from that list, unique entities are also extracted. The evaluation of approaches indicates that the proposed approach advances the state of the art in requirements entity extraction.

The remaining research paper has the following sections. Section II presents the approaches available in the literature for entity or terminology extractions. Section III consists of the background of the two parsing techniques, namely constituency parsing and dependency parsing. Section IV states the methodology, including dataset preprocessing, manual entity annotation, entity extraction using parsers, and a hybrid approach, and the evaluation metrics used to evaluate the results. In Section V results are discussed and presented. Section VI is about the external and internal threats to the validity. Section VII concludes the research paper and presents the future opportunities.

## Related work

In this section, approaches used in the literature for extracting entities, terminologies, and glossary terms from software requirements are discussed. These approaches include rule-based or statistical approaches and learning-based approaches.

### Rule-based or statistical approaches

Entity extraction is used to identify term aliases from requirements specifications, which may cause problems in understanding the requirements. POS tagging is applied on the requirements, and entities are extracted based on noun tags. Syntactically and semantically related term pairs are identified by using a combination of the approaches, including latent semantic similarity, tag-based similarity, transitive similarity, and co-location similarity. As a result, a cluster of entity-term aliases is produced [9].

The approach presented in [9] is later extended, and a more efficient solution is proposed, which results in an extended list of alias terminologies. The Latent Semantic Analysis (LSA) model is improved by adding semantic compression. The tag-based similarity approach is improved by adding tag-equivalence-based compression. WordNet-based analysis is used to eliminate false positive results. The process of cluster generation of aliases is also improved [12].

Glossaries are helpful for understanding the requirement terms and removing ambiguities and vagueness of the concepts. Clustering of these glossary terms groups related terms. The approach presented in [20] differs from existing work, as it does not provide a flat list of terms; instead, it makes clusters by relevance. Moreover, the approach is evaluated based on two industrial case studies from different domains. The terms are extracted through a noun chunking pipeline along with some heuristics. For clustering of the terms, the Expectation Maximization (EM) algorithm is used. An automated, mathematically based procedure, the Bayesian Information Criterion (BIC), is used for choosing the number of clusters. Later, the research is extended in [15] by computing the syntactic and semantic relevance between terms using state-of-the-art methods. The clusters of these relevant terms are produced by using three clustering algorithms: K-means, Agglomerative Hierarchical, and EM (performs best). The experiment is conducted on three case studies, and the perception of subject matter experts is also included. The evaluation indicates that for requirements documents, this approach gives 20 percent more accuracy in terms of recall when compared with generic term extraction tools, while the precision is comparable.

An automated hybrid approach to extract the most relevant and domain-specific glossary terms is proposed in [11], which is composed of linguistic terms extraction and statistical filtering. These technical glossary terms are the noun phrases. Linguistic extraction is based on the Natural Language Toolkit (NLTK), and it is used in the process of tokenization, POS tagging, chunking, and lemmatization. Relevance is computed by calculating the number of requirements in which the candidate term is present, and specificity is computed by doing a comparison of the number of occurrences with a non-domain-specific text corpus. The evaluation measures indicate that careful application of linguistic and statistical approaches improves recall [11].

Requirements-related knowledge is important for understanding the requirements of a specific domain, particularly when an analyst does not have relevant domain knowledge. It helps classify the requirements into functional and non-functional categories. During requirement elicitation, the real-time snippet is matched with the documented repository. The exact matched terms are extracted. The relevance of terms with respect to context is measured by calculating lexical association using generative models (based on Weighted Finite State Transducers (WFSTs) and statistical Language Models (LMs)) and discriminative models (based on Kernel methods and Support Vector Machines (SVM)). They are used to extract and classify the most related snippet [13].

To understand the problem domain, terminologies are extracted from natural language requirements using natural language processing (NLP) techniques and ML algorithms. Requirements terms are extracted based on noun phrase identification by applying a constituency parser using spaCy. An algorithm using spaCy and two heuristic rules is used to extract the requirement terms. These terminologies are ranked with respect to relevance by using a TextRank algorithm based on unsupervised graphs, and features are extracted for feature modelling [10].

Entity term identification is also important during the extraction of domain elements for generating class diagrams from software requirements. A language model is constructed to identify the type of structure of the sentence based on the verb patterns proposed by Hornby. Type dependency is generated by the Stanford Parser API, which is used to identify the semantic relationships of words in a requirement statement to identify the sentence structure. POS tagging is done for each sentence to extract entity terms based on noun phrases, and heuristics are applied to identify entity classes and attributes. Different types of relationships (including association, generalization, and aggregation) are identified based on rules, and a class diagram is generated. The evaluation metrics for the class diagram used in the experiments are correctness, completeness, and redundancy [18].

### Learning-based approaches

Glossary terms are not only extracted from software requirements but also from source code and the documentation of the project. The concept extraction process consists of the extraction of a seed term, the extraction of an extended term, and the merging of an alias. Initial seed terms are extracted from the source code and sentences using heuristics. A semi-supervised learning method, Long Short-Term Memory-Conditional Random Fields (LSTM-CRF), is used iteratively to extract more candidate terms from the sentences. Aliases of the same concept are detected from the candidate terms. The lemmatization of the candidate terms is performed, and then morphological synonyms are identified and merged. Similarly, abbreviations are also detected and merged [21].

To address the challenges of requirements entity extraction, including domain specificity and extensive effort in labelling, a semi-automated approach called Requirement Entity Extraction (RENE) is proposed. It is a transfer learning approach, and its main components are the LSTM language model and the LSTM-CRF model. The LSTM language model is trained on a general corpus and fine-tuned on unlabelled requirement sentences. The LSTM and embedding layers from the LSTM language model are transferred to the LSTM-CRF model. Each requirement entity is assigned a word sequence using labelled requirements, and a BIO (Beginning, Inside, Outside) tag is assigned to each word in the word sequence. The CRF layer is trained using sequence tagging of words. The output is the automated extraction of requirement entities in BIO format. This approach requires labelled requirements for sequence tagging, which is a labour-intensive task [6].

Machine learning approaches are used for information extraction tasks. The spaCy provides an NLP pipeline that includes neural network-based components, and BERT is a transformers-based model that uses bidirectional encoder architecture. These models are pre-trained on a generic dataset and are used for the information extraction tasks. The dataset is BIO tagged to extract entities, including name, location, facility, and relation types. The sentences are extracted from paragraphs containing these tags. Relationship types are extracted using dependency parsing in spaCy. For domain-specific tasks, the models are trained on a domain-specific dataset to extract the domain-specific entities and to classify those entities to serve the task of information extraction [8].

The NER approach is used to identify general real-world objects, namely location, person, time, date, and organization. Now, NER approaches are also used to identify domain-specific entities in software requirements. The dataset used in the study consists of requirements from DOORS (Dynamic Object-Oriented Requirements System) software. Entities are categorized into ten classes, and the BIO tagging scheme is used for manual annotation of the requirements. The experiment is conducted using three approaches, including ML-CRF, Conditional Maximum Entropy Model (C-MEM), and Bidirectional Long Short-Term Memory (BiLSTM). The results indicate that the ML-CRF approach gives the best result, contrary to the deep learning-based BiLSTM approach, and the reason is the deep learning-based approaches require a larger dataset for training to give the best performance [7]. Later, the study is extended, and two more datasets, including SER (Software Requirement Entities) and RQA (Requirement Quality Assistant), are used. A comparison of ML-CRF with deep learning-based techniques, including BERT, RoBERTa, and ALBERT, indicates that BERT performs best in the case of DOORS and SRE datasets, while ML-CRF performs best for the RQA dataset [1].

## Background of parsing techniques

In our research experiments, we have applied two parsing techniques, i.e., constituency and dependency parsing, so it is important to know how these parsers work. This section shows technical details of the constituency and the dependency parsers.

### Constituency parsing

There are three main approaches for constituency parsing, which include: Transition-based methods [22- 23], sequence-based methods [24- 25], and chart-based methods [26 - 28]. The approach used in this research for constituency parsing is the chart-based method presented in [27]. The architecture of the constituency parser is based on (1) a self-attentive encoder and (2) a decoder mechanism. The encoder in this architecture is an improved version over the previously used LSTM-based encoder. The encoder reads the input sentence, converts it into vector format, and helps in identifying the important compositions of the sentence for parsing. It considers both the positional relationships among nearby words and the broader sentence context to learn meaningful representations. This contextualized representation helps the parser capture the compositional structure of the sentence and improve parsing performance. For unseen or rare words, learning representations from their constituent parts can further support accurate parsing. There are 8 layers in the self-attention mechanism, and each layer is further divided into two sublayers (multi-head attention mechanism and position-wise feed-forward sublayer). Each sublayer is followed by a layer normalization. In the multi-head attention mechanism, each word incorporates information from 8 locations, while in the position-wise feed-forward mechanism, the same mechanism is applied to each word, and no word takes any information from any other word. The decoder part converts the vectors generated in the encoder mechanism to create a labelled parse tree [27]. The decoding mechanism is based on the CKY-style inference algorithm that selects the constituent tree based on the highest scores [27, 29].

### Dependency parsing using spaCy

SpaCy is a neural network-based open-source library that is useful in different NLP tasks such as POS tagging, dependency parsing, lemmatization, and rule-based matching [8, 30]. A dependency parser is used to understand the syntax and semantics of a sentence [31]. SpaCy uses a transition-based dependency parsing technique [8]. Transition-based parsing does not depend on formal grammar; instead, it is based on the training on Treebank data. For dependency parsing, it uses a state machine model and scores the possible next transition to generate a dependency tree. The state machine is termed as a transition system, and the possible next actions are termed as transitions [32]. The words in a dependency tree are connected by an explicit relation called a dependency relation. In a dependency relation, one word represents a head word, while other words are labelled as dependents [33]. Nodes of the dependency tree are single words so that a dependency relationship can be built between these words [34]. Arrows are used to represent the dependency relations. These arrows have a clear label indicating the dependency type. The dependency type represents the type of relationship between words, for example, nsubj, obj, etc. The dependency structures produced by the dependency parser differ from the phrase structures. The dependency structures represent relations in the form of functional categories such as subject, object, and modifier, while phrase structures are represented in the structure categories such as verb phrase (VP) and noun phrase (NP) [32].

## Methodology

The methodology used for conducting this research consists of five different stages. These are (A) Data Collection, (B) Data Preprocessing, (C) Manual Entity Annotation, (D) Extracting Entities Using Parsers, (1) Entities Extraction Using Constituency Parser, (2) Entities Extraction Using Dependency Parser, (3) Entities Extraction Using Hybrid Approach, and (E) Evaluation Metrics. The overall methodology is presented in Fig 1.

**Fig 1:**
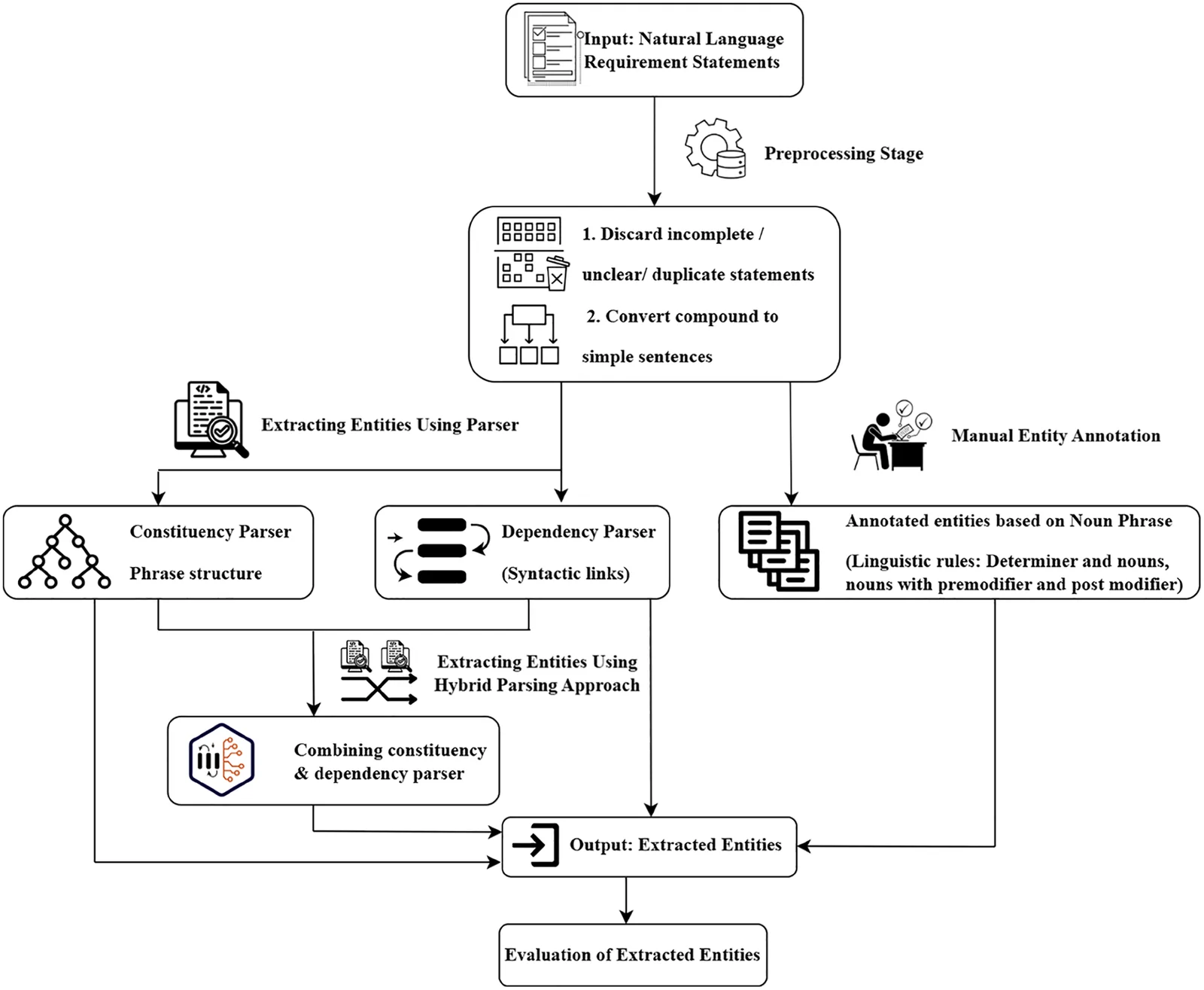
Overview of methodology to extract requirement entities.

In the methodology, we describe the source of data and the preprocessing of the software requirements statements. Then we explain the process of manual entity annotation from software requirements, in which entities are identified and annotated as noun phrases. We independently apply two parsers to extract noun phrases following the manual entity annotation. Fig 1 shows that the constituency parser and dependency parser are applied to extract noun phrases from software requirements. Next, a hybrid approach combining the results of both the constituency and dependency parsers is applied. The final output is a list of extracted entities. These entities are evaluated based on manually annotated entities, which are considered the gold standard. The evaluation metrics used to evaluate the performance of the approaches are also discussed in this section.

### Data collection

There is a scarcity of domain-specific, open-access corpora of software requirements. So, a corpus is developed based on the software requirements derived from multiple independent academic software engineering projects developed and evaluated under subject matter instructors. The healthcare domain is considered for the experiments, and the functional requirements of a total of 9 projects are used in the research, which ensures the diversity in requirements phrasing. Table 1 shows the categories of different projects, including Hospital Management System, Online Consultation, Vaccine Management System, Donation Systems, and Skin Disease Detection System, and their corresponding projects.

**Table 1:** Project categories.

Project Category	Projects
Hospital Management System	P1, P6
Online Consultation System	P3, P5
Vaccine Management System	P2
Donation Systems	P4, P7, P8
Skin Disease Detection System	P9

### Data preprocessing

The rules used for data preprocessing are based on prior research in the requirements engineering domain. The requirements statements, in the form of a single sentence, are used in the study. The requirements are converted from compound statements to single statements to ensure consistent analysis [1, 16]. The incomplete and duplicate requirement statements are discarded [17]. Requirement statements with a simple sentence structure are included. The complex statements that are difficult to understand are converted to simplified sentence structures [17, 18]. These preprocessing steps do not remove or alter semantic content. They separate compound requirements into simpler, self-contained units while preserving their original meaning for analysis. Initially, there were 766 requirement statements consisting of compound, incomplete, and duplicate statements. There were 82 incomplete and duplicate requirement statements. The duplicate requirements were from the account creation and login modules. There was a total of 191 compound requirement statements, which were converted to atomic statements. After applying all these rules, the total corpus comprises 875 requirement statements collected from 9 software projects in the healthcare domain. The project-wise distribution of requirements is mentioned in Table 2.

**Table 2:** Project-wise distribution of software requirements.

Project	P1	P2	P3	P4	P5	P6	P7	P8	P9	Total
**Requirements**	183	136	62	162	114	117	40	33	28	875

### Manual entity annotation

The goal of this step is to manually annotate entities based on empirical heuristics. Entities can be single-word entities representing real-world objects and multiword entities in the form of compound nouns and noun phrases [9, 18]. The focus of this research is to annotate all possible entities, including single-word entities and multi-word entities.

Noun phrases consist of a head noun, determiners, and modifiers, or the elements that complement another element in the phrase [35]. In natural language, there are two types of noun phrase modifiers: 1) occurs before the head noun (premodifier) and 2) occurs after the head noun (postmodifier) [36]. Comparative and superlative adjectives are commonly used as modifiers of nouns. There are simple-to-complex-level premodifiers of a noun, which include adjectival phrases, noun phrases, verbal phrases, prepositional phrases, and adverbial phrases [37]. There are two main categories of postmodifiers in noun phrases. The first category is clausal postmodifiers, which include relative clauses, appositive clauses, and non-finite clauses. The second category of postmodifiers is phrasal postmodifiers, and it includes adjective phrases, appositive noun phrases, prepositional phrases, and non-appositive noun phrases [38]. Complex noun phrase structure also includes binominal noun phrases, which consist of two nouns separated by a preposition. It is important to understand the head of the construction in the case of a binominal noun phrase, which is the second noun [39]. The noun phrase extraction exhibits two structural phenomena described in linguistic theory: recursion and structural iteration. The recursion has the embedded constituents inside the constituents of the same type. It is based on the hierarchical structure of the sentence. The iteration is the repeated addition of the same structural type as the existing one. Iteration is observed at the same structural level and does not require a hierarchy [40]. Our extraction of noun phrases is based on these concepts.

In our study, individual nouns are extracted using a unified noun phrase decomposition strategy, where determiners are removed from a noun phrase in a determiner-noun construction. Compound nouns are extracted as complete compounds and as valid meaningful sub-spans. Postmodifiers are predominantly based on recursive structure with a maximum depth of level 2. They are decomposed hierarchically, terminating at the individual nouns extraction that serves as the lowest span level. Premodifiers are treated as iterative structures because true recursive premodifiers are relatively uncommon. Therefore, all the modifiers preceding the head noun are considered at the same level without a hierarchy.

Two annotators, one having background knowledge in the natural language processing domain and the other having a background in the requirements engineering domain, independently annotated the noun phrases based on the understanding of head nouns, determiners, compound nouns, premodifiers, and postmodifiers in noun phrase annotation rules. The agreement is based on the exact matching of the span boundaries by both the annotators. A candidate span was considered a negative case when an annotator extracted it but did not identify it as a valid noun phrase. In case of a disagreement between the two annotators, a third annotator who is a linguistic expert serving as adjudicator resolves the disputes by evaluating the candidate span according to the noun phrase annotation rules. When the third annotator does not agree with either annotator, the candidate span is discarded.

To verify the inter-annotator agreement, Cohen’s Kappa statistics are applied [41]. Kappa statistics are calculated by applying Equation 1. In Equation 1, k represents kappa, Po represents observed agreement, and Pc represents chance agreement. Equations 3 and 4 are used to compute Po and Pc respectively. The values of Po and Pc are passed in Equation 1 to compute the value of k.


$$\begin{array}{c} \kappa =\frac{P_{o}-P_{c}}{\text{1}-P_{c}} \end{array}$$ (1)

$$\begin{array}{c} N=a+b+c+d \end{array}$$ (2)

In Equation 2, “a” represents that both annotators identified the candidate span as valid noun phrase (agreement positive), “b” represents that only the first annotator identified a candidate span, “c” represents that only the second annotator identified a candidate span, and “d” represents candidate spans rejected by both annotators (negative agreement). The values of a, b, c and d are 5416, 11, 14 and 76 respectively. The value of Equation 2 is used in Equations 3 and 4 as a denominator to calculate the observed and chance agreement.


$$\begin{array}{c} P_{o}=\frac{a+d}{N} \end{array}$$ (3)

$$\begin{array}{c} P_{c}=\frac{\left(a+b)(a+c)+(c+d)(b+d\right)}{N^{\text{2}}} \end{array}$$ (4)

The value of kappa is 0.86, which shows that there is substantial agreement between the two annotators. The 25 candidate spans on which the annotators disagreed were subsequently reviewed by the third annotator, of which 19 were judged to be valid noun phrases and included in the final dataset. The 76 candidate spans rejected by both annotators were discarded. Our dataset contained all the mentioned types of premodifiers, as well as phrasal postmodifiers. Among postmodifier clauses, only relative clauses and appositive clauses were found in our dataset. For example, in the multiword entity “record of a doctor”, the record is the head noun, and doctor is its postmodifier. The project-wise distribution of the annotated entities is shown in Table 3. Many entities appear multiple times in the requirements, so it is important to identify unique entities for analysis. Unique entities for each project are derived, and these are also displayed in Table 3.

**Table 3:** Number of manually annotated entities and unique entities from each project.

Project	P1	P2	P3	P4	P5	P6	P7	P8	P9	Total
**Entities**	1060	934	379	1168	619	659	220	202	194	5435
**Unique Entities**	253	430	190	334	287	291	104	105	129	2123

### Extracting entities using parsers

In this section, baseline approaches and entity extraction using different parsing methods are discussed. Three parser-based techniques are presented in the subsequent subsections: (1) Entity Extraction Using Constituency Parser, (2) Entity Extraction Using Dependency Parser, and (3) Entity Extraction Using Hybrid Approach.

A pretrained BERT-based chunking model, bert-english-uncased-finetuned-chunk, obtained from the Hugging Face Transformers library, is employed as a neural baseline to identify noun-phrase chunks from the requirements. The model performs token-level chunk classification using BIO-based chunk labels and reconstructs syntactic chunks from the predicted labels.

The model primarily identifies base noun phrases, compound nouns, and noun phrases containing premodifiers. However, its BIO-based chunk representation does not directly support the systematic extraction of postmodifier-based spans required in this study. An additional module is applied to extract individual nouns and relevant sub-spans within the predicted noun-phrase chunks. A normalization step is also applied to standardize apostrophe-containing constituents. The model is used without domain-specific fine-tuning to provide a pretrained BERT-based neural baseline for comparison with the proposed parser-based approach.

To contextualize the performance of the constituency parsing-based approach evaluated in the study, a parser-based baseline reported in the literature is used as the baseline.

**NP Method** [10] is a baseline approach that extracts requirements terminologies using a constituency parser, which is implemented using spaCy. A constituency parse tree is generated for each sentence, and the requirements terminologies are extracted based on noun phrases. The NP Method is evaluated on smart home requirements dataset, which is based on user stories. The results of the requirements terms extractions are presented in terms of precision, recall, and F1-score.

We have implemented the NP-based requirement-term extraction approach on the healthcare requirements dataset. For a direct comparison with the baseline, full noun phrase spans and their sub-spans are not treated as separate entities during evaluation. Single word and multiword noun phrases are considered according to the baseline extraction criteria.

The baseline constituency-based requirement-term extraction approach is then modified by removing the stop word removal and lemmatization steps and extended to identify a broader set of relevant noun phrase spans. The entity extraction task requires additional relevant sub-spans that cannot be directly extracted by the constituency parser. Therefore, additional extraction rules are incorporated to identify such spans and improve the coverage of NP-based entity extraction.

The configurations of the constituency and dependency parsers, along with their associated NLP models, are summarized in Table 4. The entity extraction procedures based on constituency and dependency parsing are described in the following subsections.

**Table 4:** Configuration details for constituency and dependency parsers.

Component	Configuration
Execution environment	Google Colab
Runtime	Default Colab runtime
Programming language	Python (Version 3.12)
NLP framework	spaCy (Version 3.8.2)
Constituency parser	Berkeley Neural Parser (Benepar)
Benepar model	benepar_en3 (Version 0.2.0)
Dependency parser	spaCy dependency parser
spaCy language model	en-core-web-lg-3.8.0
Pandas	Version 2.2.3
Transformers	Version 4.38.2
Tokenizers	Version 0.15.2
Input format	CSV

#### Entity extraction using constituency parser.

This section covers the workflow of the constituency parser, the algorithm describing the constituency-based entity extraction process, the formation of a constituency tree, additional modules applied for parsing, and grammatical patterns identified by the parser. Fig 2 indicates that a text input is provided to the spaCy pipeline and a pre-trained language model is loaded. In this experiment, we have applied Berkeley Neural Parser (Benepar) as a pipeline component integrated into spaCy for constituency parsing on the software requirements dataset of the healthcare domain. The actual constituency parsing mechanism starts with the self-attentive encoder and a decoder. The encoder converts the input into a vector format. Whereas the decoder generates output in the form of a parse tree, and noun phrases are extracted from the parse tree. Algorithm 1 explains the process of entity extraction using a constituency parser.

**Fig 2:**
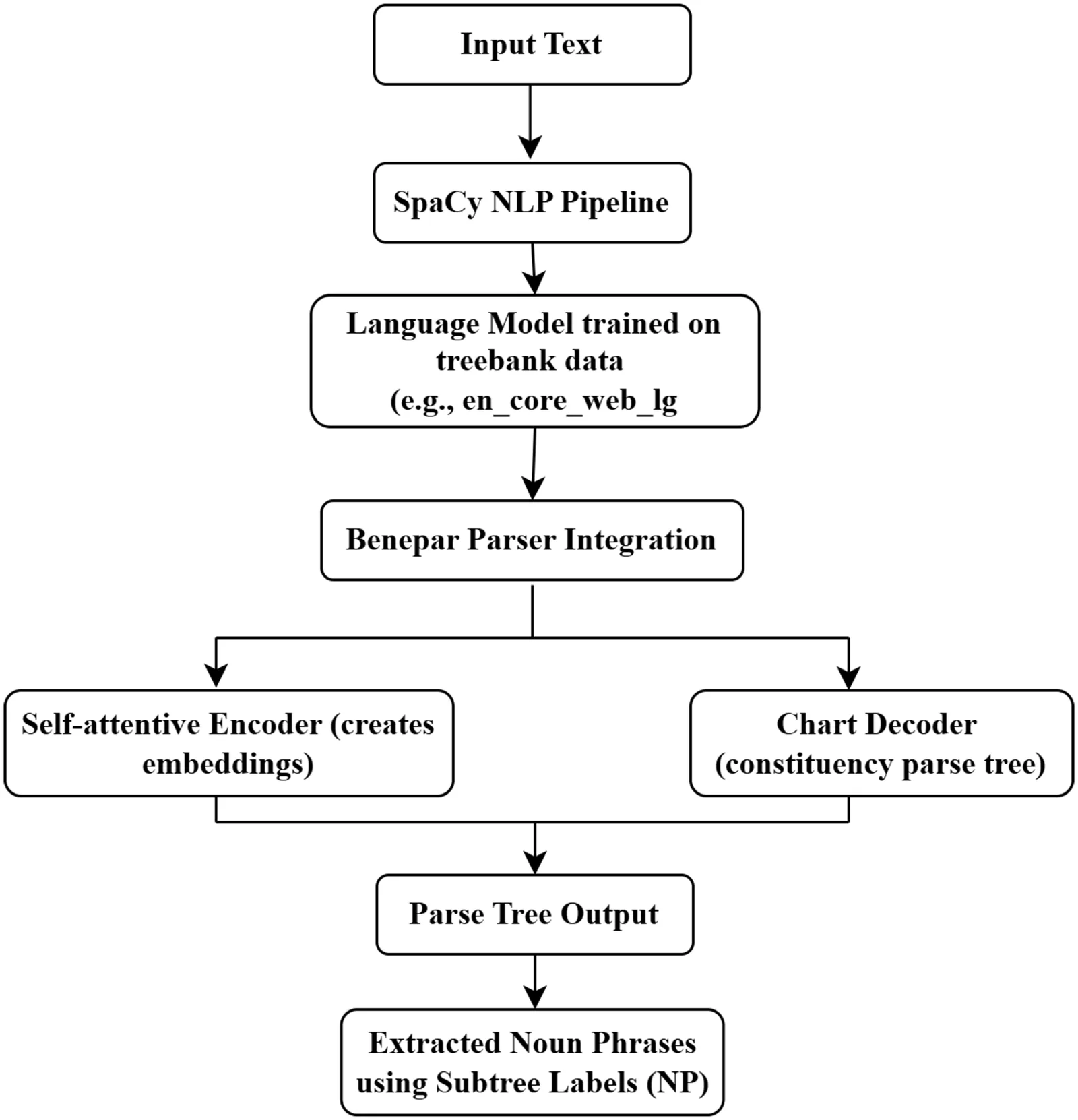
Working flow of constituency parser.

**Algorithm 1: Constituency-Based Requirement Entity Extraction.**

**Input:** CSV file (R.CSV) containing software requirements

**Output:** CSV file containing constituency parse trees and extracted entities

1 Load spaCy model en_core_web_lg and Benepar constituency parser.

2 Read R.CSV into dataframe D.

3 Initialize columns “Constituency Parse” and “Constituency” in D

4 Define OMIT_WORDS ← {“system,” “the,” “a,” “an,” “their”}.

5 for each requirement r ∈ D do

6  doc=NLP(r)

7  initialize ALL_PHRASES = ∅

  // Basic constituency-based NP extraction

8  for each NP constituent c in doc do

9   p = CLEAN_PHRASE (text(c), OMIT_WORDS)

10   if p ≠ ∅ then

11    Add p to ALL_PHRASES

12   end if

13  end for

   // Refinement using spaCy noun chunks

14   foreach noun chunk nc ∈ doc do

15    tokens=tokens in nc with POS ∈ {NOUN, PROPN, ADJ}

16    Remove tokens belonging to OMIT_WORDS

17    if tokens ≠ ∅ then

18     term= concatenate(tokens)

19     Add term to ALL_PHRASES

20    end if

21   end for

  // Extraction of compound nouns and two-word subphrases

22  for each token n ∈ doc do

23   if POS(n) = NOUN then

24    c = compound modifiers of n occurring on its left

25    Remove tokens in OMIT_WORDS from c

26    if length of c > 1 then

27     Add concatenate(c) to ALL_PHRASES

28     for each adjacent token pair in c do

29      Add concatenate(pair) to ALL_PHRASES

30     end for

31    end if

32   end if

33  end for

  // Extraction of individual nouns

34   for each noun token n ∈ doc do

35    if POS(n) = NOUN and n ∉ OMIT_WORDS then

36     Add text(n) to ALL_PHRASES

37    end if

38   end for

  // Possessive-based deduplication

39 FILTERED_PHRASES ← REMOVE_POSSESSIVE_SUBPARTS(ALL_PHRASES)

  // Store results

40 Store constituency parse tree of doc in “Constituency Parse”

41 Sort FILTERED_PHRASES

42 Store FILTERED_PHRASES in “Constituency”

43 end for

44 Export D to CSV.

Lines 1–3 load the pre-trained spaCy language model and Benepar constituency parser, read the requirements into a dataframe, and output columns are initialized. Lines 4–7 iterate through each requirement, generate its linguistic representation, and initialize ALL_PHRASES with an empty set to store extracted phrases. Lines 8–13 perform the constituency-based extraction by identifying noun phrase constituents from the parse tree, cleaning the extracted phrases by removing the omit words, and adding the resulting phrases to ALL_PHRASES. Lines 14–21 process spaCy noun chunks and retain noun, proper-noun, and adjective tokens. Lines 22–33 extract compound nouns and adjacent two-word noun subphrases to capture additional entity spans. Lines 34–38 individual nouns as atomic entities are extracted. Lines 39–43 handle possessive phrases, the resulting entity set is sorted, and the constituency parse tree and extracted entities are stored in the dataframe. Finally, line 44 exports the processed requirements and extracted entities to a CSV file.

A constituency tree is used to represent a hierarchical form of sentence composition [42]. The phrases are extracted using a constituency parser, and these may be noun phrases, verb phrases, and prepositional phrases. The resulting tree represents these phrases in a hierarchical form [33]. From these different phrases, noun phrases are considered in this research. The nodes of the tree represent the span of words, and links are used to show the relations [34]. These spans have internal structure, having nodes, for example, S, NP, DT, NN, VP, MD, VB, and JJ. The labels of these nodes are based on the Penn Treebank POS tag set [43]. An example of a constituency parse tree is shown in the Fig 3.

**Fig 3:**
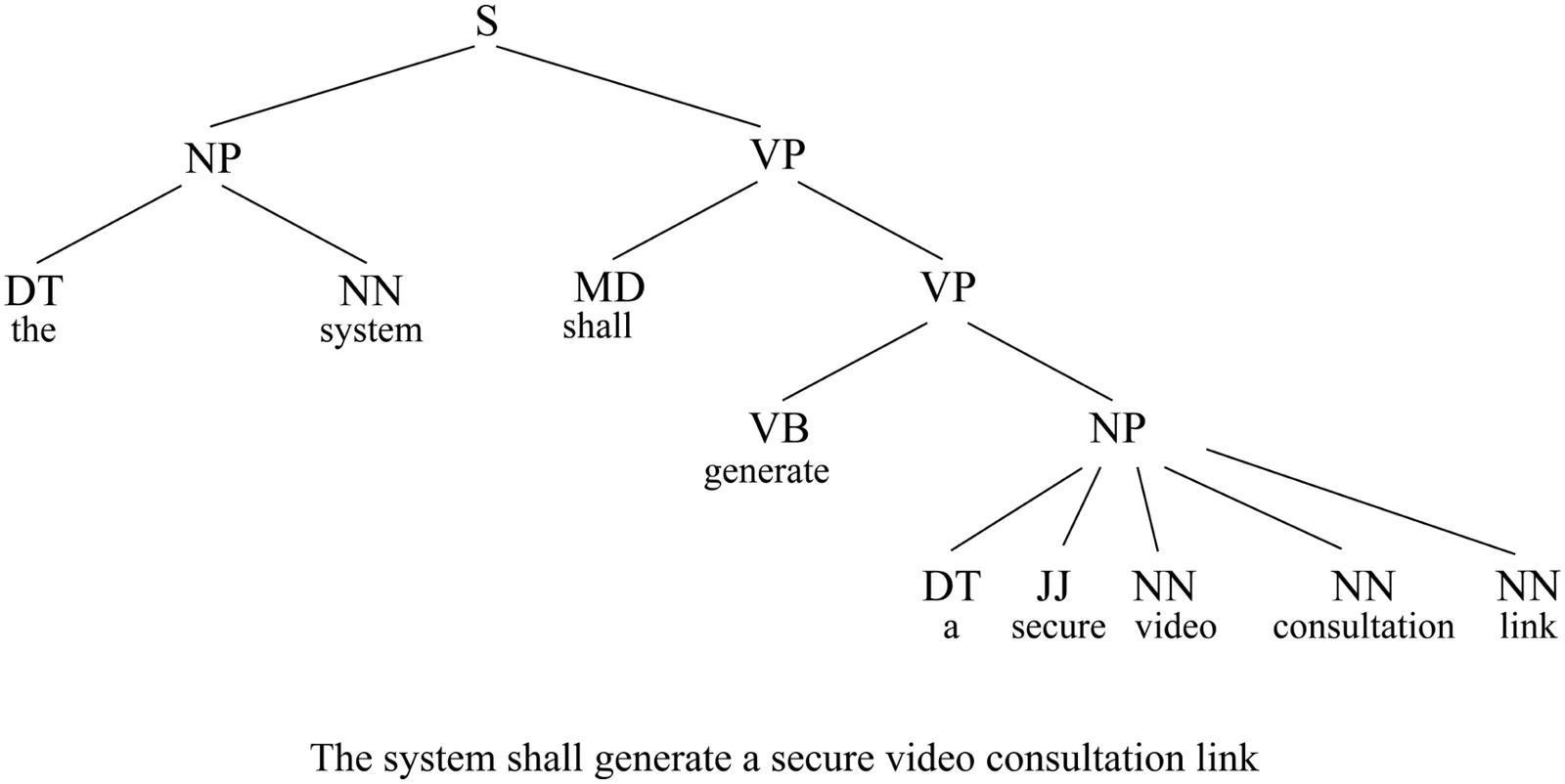
Example of a constituency parse tree.

The example sentence is “The system shall generate a secure video consultation link.” The constituency parse tree of the overall sentence comprises a span of NP followed by a span of VP. The base noun phrases in this example are “the system” and “a secure video consultation link”, whereas its compound noun phrases are “video consultation link”, “video consultation”, and “consultation link.” The individual nouns are “system”, “video”, “consultation”, and “link.” All these noun phrases are entities and are useful in different NLP tasks.

We have extracted the noun phrases that contain base noun phrases, noun phrases with premodifiers and noun phrases with postmodifiers, compound noun phrases, and individual nouns in our experiment. The constituency parser primarily extracts noun phrases, including base noun phrases, compound nouns, and noun phrases with premodifiers and postmodifiers. According to our noun phrase extraction strategy, premodifiers are treated as iterative structures and postmodifiers are based on recursive structures due to its hierarchical tree structure. To extract all possible noun phrases, we have developed an additional module to decompose multi-word sequences into sub-spans. A module that traverses the tree to create individual nouns has also been developed, and fine-grained individual nouns are created by applying the module. Another problem using the constituency parser is that it does not handle the possessive constructions due to the tokenization of the apostrophe. A normalization step is applied to handle apostrophes in constituent parts and remove the possessive duplications. For example, in the phrase “doctor’s profile”, the parser was generating redundant output like “doctor’s”, “doctor’ s”, and “doctor” as separate noun phrases. So, by applying the normalization step, this error is removed. To convert these noun phrases into entities, the determiners (articles including ‘the’, ‘a’, and ‘an’) are removed from the extracted noun phrases.

The tagging and grammatical structure used in the constituency parsing can be generalized to form rules [42]. A total of 13 categories of patterns are identified by the constituency parser from our dataset. The POS tagging used in the example patterns is based on the Penn Treebank POS tag set [43]. Table 5 contains categories of patterns, example patterns from a specific category, and extracted example phrases from the requirement statements.

**Table 5:** Pattern category with example patterns and example phrases extracted by the constituency parser.

#	Pattern Category	Example Pattern	Example Phrase
1	Simple NP	NN-NN, JJ-NN	user role, appropriate message
2	Complex NP	DT-JJ-NN-NN-NNS, RB-JJ-NN	all ongoing blood donation campaigns, currently available news
3	Prepositional Phrase-Based NP	JJ-NN-IN-NN-NN-NN, NNS-IN-DT-NN-NN	detailed information of blood donation center, details of a staff member
4	Participial NP	DT-VB-NNS, VBN-JJ-NNS	all recorded expenses, recommended seasonal vaccines
5	Participial-Prepositional NP	JJ-NN-IN-VBN-NN, NN-IN-VBN-NN-NNS	online payment for booked appointment, size of uploaded image files
6	Infinitival NP	NN-TO-VB-DT-NN-NN, NN-TO-VB-NN-NNS	option to make a blood request, mechanism to report notification issues
7	Infinitival-Prepositional NP	NN-IN-NNS-TO-VB	list of doctors to patient
8	Participial-Infinitival NP	DT-VBN-NN-TO-VB	any failed step to login
9	Multiple Participial Chains	NN-VBN-IN-DT-VBN-NN, NN-VBN-IN-DT-VBN-NN-NN	quantity required for each selected vaccine, account associated with the provided email address
10	Complex NP with a Relative Clause	JJ-NNS-WDT-VBP-NNS, NNS-WP-VBZ-NN-NN	new campaigns that match preferences, donors who fulfil platelet requirements
11	Possessive NP	NN-POS-NN-NNS, NN-POS-NNS	doctor’s working hours, patient’s details
12	Prepositional-Possessive NPs	NN-POS-NN-IN-NN-NN	user’s eligibility for blood donation
13	Participial-Prepositional-Infinitival NP	NNS-TO-VB-NN-NNS-VBN-IN-NN-NNS	filters to refine search results based on user ratings

#### Entity extraction using dependency parser.

This section covers the workflow of the dependency parser, the algorithm describing the dependency-based entity extraction process, the formation of a dependency tree, additional modules applied for dependency parsing, and grammatical patterns identified by the parser. Fig 4 indicates that a text input is provided to the spaCy pipeline and a pre-trained language model is loaded. The dependency parser assigns dependency labels, and a dependency tree is created. To extract the noun phrases from our dataset of software requirements using dependency parsing, a built-in base noun phrase iterator in spaCy is used to traverse the dependency tree.

**Fig 4:**
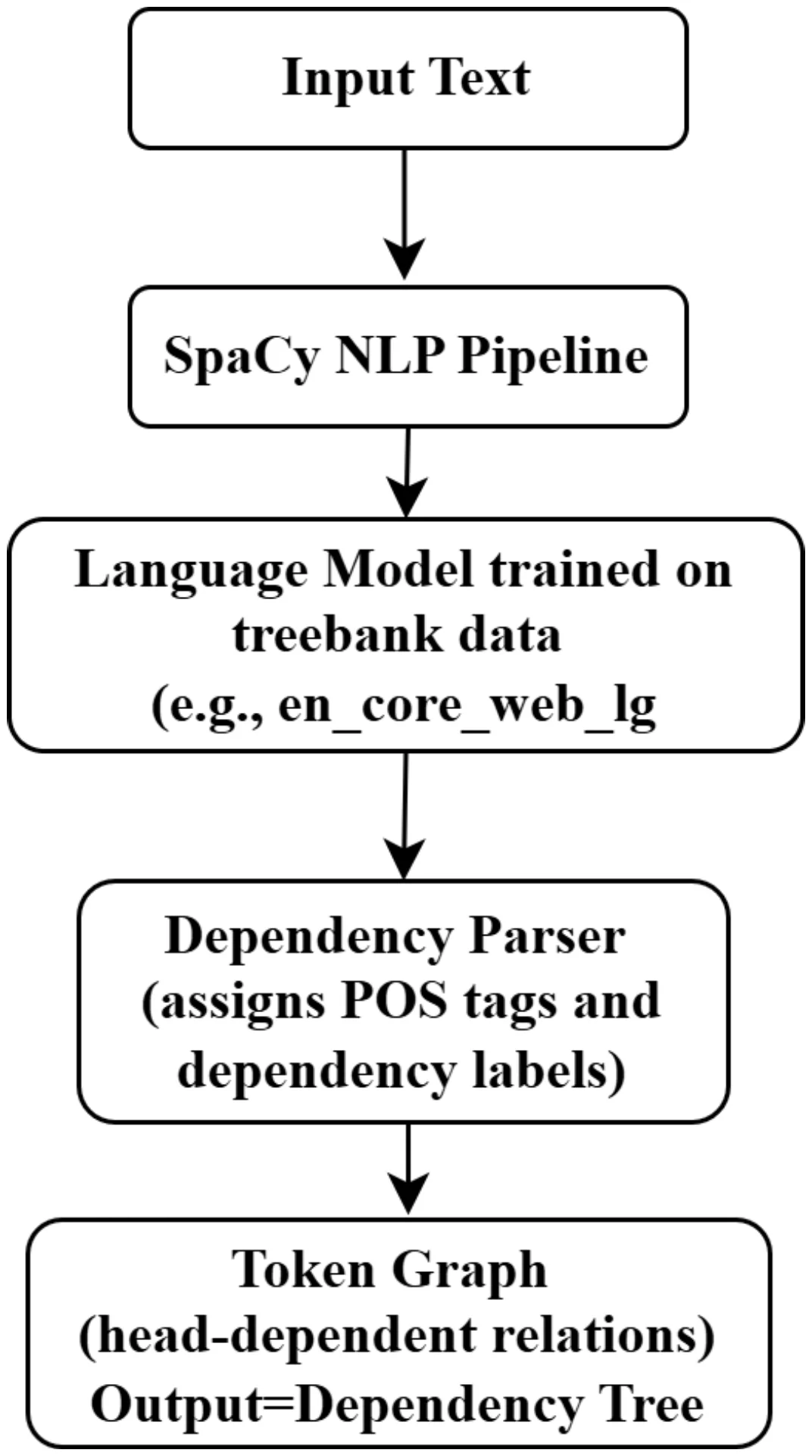
Working flow of dependency parser.

The algorithm 2 explains the process of entity extraction using dependency parser. Lines 1–3 load the pre-trained spaCy language model, read the requirements into a dataframe, and initialize the dependency output column. Lines 4–6 iterate through each requirement, generate its linguistic representation, and initialize the set (P) for storing the extracted phrases. Lines 7–12 after extracting noun chunks and possessive constructions, the resulting phrases are added to (P) after stop-word removal. Lines 13–14 identify hyphenated terms and remove redundant fragments generated during extraction. Lines 15–19 initialize the final entity set (E) and generate additional entity spans by extracting valid suffix subphrases and the head noun from each phrase. Lines 20–22 further add individual nouns excluding stop words. Line 23 sorts the resulting entity set and stores it in the Dependency column. Finally, line 25 exports the processed requirements and extracted entities to a CSV file.

Dependency parsing is more focused on the relations between the words [33]. The relation between a noun and an adjectival attribute is an example of the type of relation extracted by dependency parsing [42]. When a sentence is parsed using a dependency parser, it generates a dependency tree as an output. An example of a dependency parse tree is given in the Fig 5.

**Fig 5:**
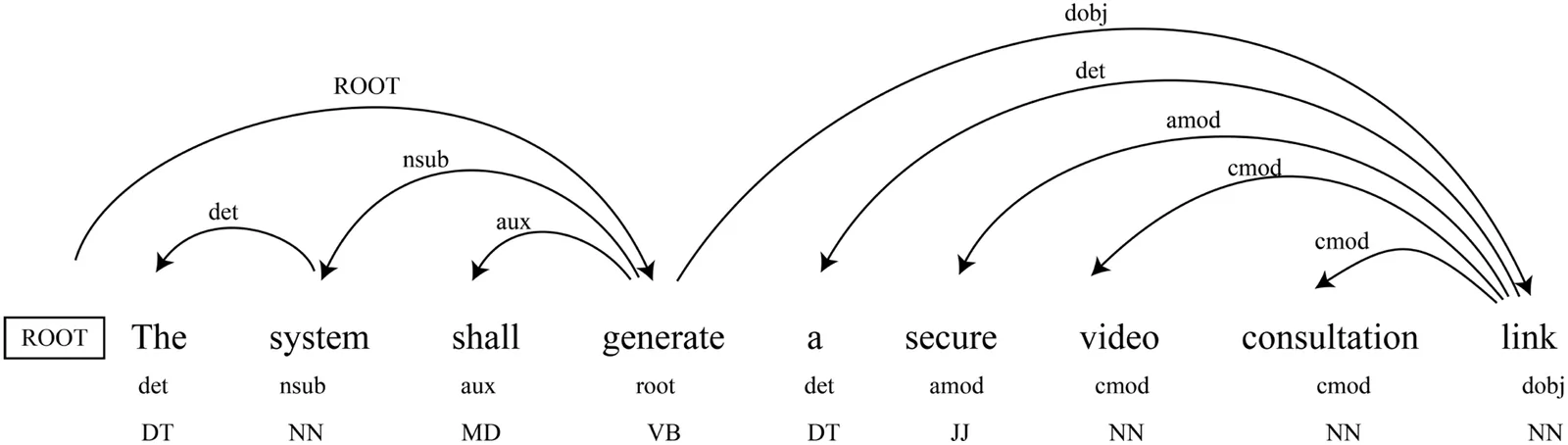
Example of a dependency parse tree.

**Algorithm 2: Dependency-Based Requirement Entity Extraction.**

**Input:** CSV file containing software requirements

**Output:** CSV file containing extracted requirement entities

1 Load the pre-trained spaCy language model (en_core_web_lg)

2 Read the requirements document R into dataframe D

3 Initialize the Dependency column in D

4 foreach requirement r∈Ddo

5  doc=NLP(r)

6  P=∅

  // Extract noun chunks and possessive phrases

7  foreach noun chunk nc∈docdo

8   Add RemoveStopWords(Text(nc)) to P

9  end for

10  foreach possessive relation (x,y)∈docdo

11   Add RemoveStopWords(ConstructPossessivePhrase(x,y)) to P

12  end for

  // Extract and refine hyphenated terms

13  Add all hyphenated terms in docto P

14  Remove redundant hyphen fragments from P

  // Generate additional entity variants

15  E=P

16  foreach phrase p∈P do

17   Add valid two-or-more-word suffix subphrases of p to E

18   Add HeadNoun(p) to E

19  end for

  // Add individual nouns

20  foreach noun n∈docdo

21   Add Text(n)to E, if nis not a stop word

22  end for

23      D[r,Dependency]=Sort(E)

24 end for

25 Export D to CSV

The tree shown in Fig 5 is divided into two parts starting from the root node. The root node is “generate”, which is a verb. “Shall” is used as a modal auxiliary of “generate”, “system” is a noun and subject of the root node “generate”, and “The” is a determiner of the subject node “system”. The other part of the tree has “link” as the direct object of “generate”. “Consultation” and “video” are clausal modifiers of “link”, “secure” is an adjective modifier of the “link”, and “a” is the determiner. The entities extracted in this sentence are “consultation”, “consultation link”, “link”, “secure video consultation link, “video”, and “video consultation link.”

As the dependency parser uses head dependant relation that captures linear modifiers, it can extract base noun phrases, and some noun phrases with premodifiers and postmodifiers are implicitly captured through dependency-based tree traversal. Additional modules are created and applied to extract compound nouns and to decompose multi-word sequences into sub-spans and individual noun tokens. Moreover, the algorithms are used to address the problem of apostrophes and hyphenated terms. To convert the extracted noun phrases into entities, the determiners are removed from the extracted noun phrases in the same way as we did in the case of constituency parsing.

The tags obtained while applying dependency parsing are based on the relationship between words. These tags are converted into POS tags to make different categories of patterns using the Penn Treebank POS tag set [43]. A total of 5 categories of patterns are identified by the dependency parser from our dataset mentioned in Table 6. Example phrases extracted from the requirement statements against the mentioned patterns are also provided in Table 6.

**Table 6:** Pattern categories with example patterns and example phrases extracted by the dependency parser.

#	Pattern Category	Example Pattern	Example Phrase
1	Simple NP	CD-NN, JJ-NNS	six characters, latest recommendations
2	Complex NP	DT-NN-NNS, NN-NNP-NNS	all chat threads, epidemic outbreak vaccines
3	Participial NP	DT-VBN-JJ-NNS, CD-VBD-NN-NNS	all scheduled virtual appointments, 5 failed login attempts
4	Possessive NP	NN-NN-POS-NN, NN-POS-NN	family member’s profile, doctor’s record
5	Participial-Possessive NP	VBN-NN-POS-NN-NN	updated center’s profile information

#### Entity extraction using hybrid approach.

The constituency parser is generally more effective in identifying the noun phrases of large spans. However, some sub-spans are not identified by the constituency parser because they are usually embedded inside a larger constituent structure. On the other hand, some noun phrases having recursive postmodifiers are not extracted by the dependency parser because it is based on a head-dependent relational structure that does not represent a full noun phrase span. But the dependency parser shows better performance on some possessive structures.

As the constituency and dependency parsers extract noun phrases based on different syntactic representations, their extraction capabilities are complementary. The limitations of one parsing technique can be overcome by the extraction capabilities of the other. Based on this observation, a hybrid extraction procedure is developed to combine the candidate entities generated by the two approaches.

The hybrid approach integrates the entities extracted from the constituency parse tree with the entities extracted based on the dependency relations. The hybrid approach also applies an exact-match-based deduplication of identical entity strings to maintain a non-redundant set of extracted entities and merges the exactly matched entities extracted by the two parsing techniques. The hybrid extraction procedure is formally represented by the Equation 5.


$$\begin{array}{c} Hybrid\;Approach\;=\;Constituency\;Parser\;U\;Dependency\;Parser \end{array}$$ (5)

### Evaluation metrics

Evaluation metrics used for this research are precision, recall, and F1-score.

Precision is calculated using Equation 6, where TP (True Positives) is the number of correctly extracted entities and FP (False Positives) is the number of extracted entities that are not present in the gold standard and extracted by the parser.


$$\begin{array}{c} Precision=\frac{TP}{TP+FP} \end{array}$$ (6)

Recall is computed using Equation 7, where FN (False Negatives) is the number of entities present in the gold dataset and missed by the parser.


$$\begin{array}{c} Recall=\frac{TP}{TP+FN} \end{array}$$ (7)

The F1 score indicates the harmonic mean of the precision and recall, and it is calculated by Equation 8.


$$\begin{array}{c} F\text{1}\;Score=\frac{\text{2}\times Precision\;\times Recall}{Precision+Recall} \end{array}$$ (8)

## Results and discussion

In this section, the pretrained neural-based approach is evaluated on the healthcare dataset. The baseline constituency-based approach (NP method) is replicated on the healthcare dataset and its results are compared with those reported for the Smart Home dataset. Then results of entities extracted by using the extended constituency parser, dependency parser, and hybrid approach are reported. The performance and error analysis based on noun phrase pattern categories is performed. The precision, recall, and F1-score project-wise and the combined results for all projects are discussed.

The pretrained neural-based approach applied to the healthcare requirements dataset achieves a precision of 84.59%, recall of 88.55%, and F1-score of 86.53%. The model identifies base noun phrases, compound nouns, noun phrases with premodifiers, and individual nouns; however, some erroneous extraction patterns are also observed, contributing to reduced precision. The model is unable to systematically identify noun phrases with postmodifiers, thereby contributing to reduced recall. A neural approach provides the advantage of using pretrained contextual representations and reduces the need to manually specify syntactic extraction rules. However, adapting such a model to the specific characteristics of healthcare requirements may require domain-specific fine-tuning and an appropriately annotated training dataset. For the BIO-based token-level chunking approach, such adaptation would require token-level annotation of the relevant spans, which can involve substantial manual annotation effort. These considerations motivate the use of parser-based approaches in this study, which rely on explicit syntactic structures and extraction rules and provide direct traceability of extracted spans to the corresponding parse structures, while avoiding model fine-tuning and its associated training overhead.

Following the evaluation of the pretrained neural-based approach, the parser-based NP Method is also evaluated on the healthcare requirements dataset. A comparison of the results of the NP method applied on the healthcare dataset with the NP Method using the smart home dataset is presented in Table 7.

**Table 7:** Summary of the performance comparison results of NP Method.

Approach	Dataset	Precision	Recall	F1-Score
NP Method	Healthcare (Our)	**85.86%**	**99.05%**	**91.98%**
NP Method	Smart home (baseline)	74.17%	80.40%	77.16%

The NP Method achieves a higher precision, recall and F1-score on healthcare requirements dataset as compared to smart home requirements. The substantially higher recall obtained on the healthcare dataset indicates that the NP method was able to recover nearly all of the entities included in the healthcare annotated dataset. The lower precision suggests that the NP method also extracted additional noun-phrase candidates that were not included in the manual annotation particularly the full noun phrase spans. The F1-score reflects the balance between the high recall and comparatively lower precision, indicating that the NP Method achieves a relatively high overall F1-score on the healthcare dataset.

The baseline approach reported relatively low precision despite achieving high recall. This may be partly attributable to the selective nature of manually annotated noun phrases in the baseline study. For example, the original study reports “motion” as a requirement term, while “track” is not included, although “track” functions as a noun and can form a noun phrase. Such cases suggest that the manual annotation may not represent an exhaustive set of syntactically valid noun phrases. Consequently, valid noun phrases identified by the parser may be counted as false positives when evaluated against the manual annotations resulting in reduced precision and F1-score of an NP-based extraction approach.

### Entity extraction

In this section, we discuss the number of entities extracted from each project and the total entities extracted from all projects using a constituency parser, dependency parser, and hybrid approach. There is a repetition of the entities extracted by the parsers and the hybrid approach, so we have also extracted unique entities. The results of overall and unique entities extracted by all three approaches from each project and from all projects collectively are shown in Table 8.

**Table 8:** Number of requirements, total entities, and unique entities extracted project-wise and their aggregate counts.

Project	Total Requirements	Entities Extracted by Constituency Parser		Entities Extracted by Dependency Parser		Entities Extracted by Hybrid Approach	
		**Total Entities**	**Unique Entities**	**Total Entities**	**Unique Entities**	**Total Entities**	**Unique Entities**
**P1**	183	995	220	959	189	1025	228
**P2**	136	874	392	821	328	925	416
**P3**	62	358	171	334	151	364	175
**P4**	162	1116	293	1017	260	1140	306
**P5**	114	595	266	535	218	598	268
**P6**	117	631	274	592	236	648	281
**P7**	40	207	93	194	78	212	96
**P8**	33	192	98	176	86	195	100
**P9**	28	183	121	169	107	193	130
**Total**	**875**	**5151**	**1928**	**4797**	**1653**	**5300**	**2000**

Table 8 shows that P1 has the highest number of requirement statements (183), but the maximum number of entities are extracted from the requirements of P4. It suggests that the maximum number of entities is not dependent on the number of requirement statements, and a fewer number of requirements of a project may have a greater number of entities. Although the number of entities extracted by different parsers and the hybrid approach varies, it is evident that both the parsers and the hybrid approach extract the maximum number of entities from P4. If we consider the project-wise extraction of unique entities, then the project P2 has the maximum number of unique entities.

If we compare the total number of entities extracted and the total number of unique entities extracted by different approaches in Table 8 with those of the total number of entities annotated manually and total number of unique entities derived manually mentioned in Table 3, it is evident that the results of the hybrid approach are very close to the gold standard. We have also extracted the unique entities cumulatively for all projects to remove the duplication across the projects. Table 9 presents the total unique entities across all nine projects.

**Table 9:** Total number of unique entities cumulatively extracted from all nine projects by different approaches.

Unique Entities Derived from Manually Annotated Entities	Unique Entities Extracted by Constituency Parser	Unique Entities Extracted by Dependency Parser	Unique Entities Extracted by Hybrid Approach
1675	1474	1201	1538

To represent the common entities and show the difference of extracted entities, we have presented a Venn diagram in Fig 6. The Venn diagram in Fig 6(A) indicates common entities annotated manually and extracted by using the hybrid approach, which are 1462. Whereas 76 unique entities are extracted by the hybrid approach, and 213 unique entities are derived manually and missed by the hybrid approach.

**Fig 6:**
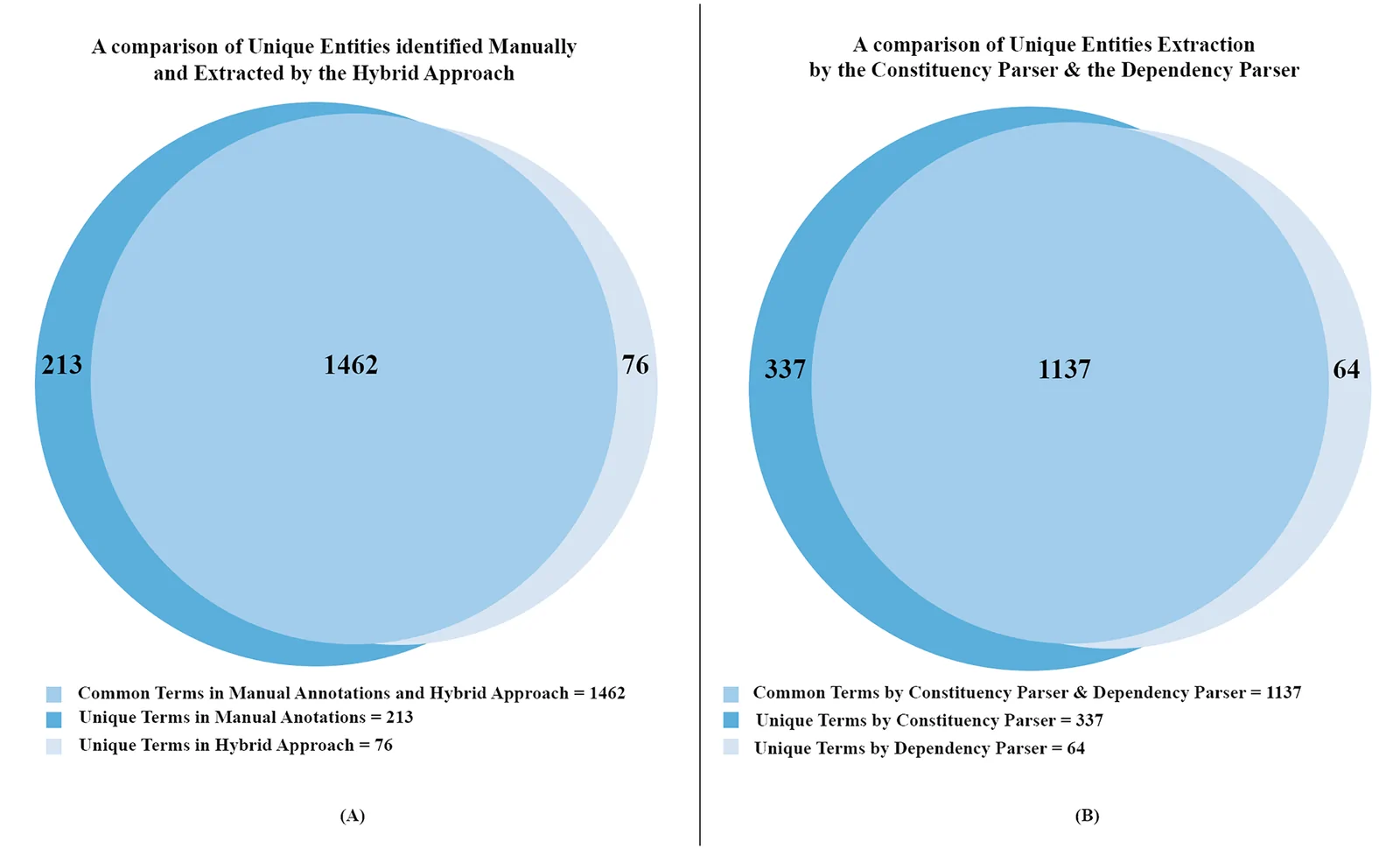
A comparison of the common and unique entities extracted by different parsers and manual annotations.

Similarly, the Venn diagram in Fig 6(B) shows a comparison between the entities extracted by the constituency and dependency parsers. There are 1137 unique entities commonly identified by both parsers. The number of entities solely identified by the dependency parser is 64, whereas the constituency parser extracts 337 unique entities. This shows the better performance of the constituency parser as compared to the dependency parser.

For example, there are two requirement statements in Table 10. In statement 1, the constituency parser extracts the entity “profile of doctor”, which is not extracted by the dependency parser. In requirement statement 2, the dependency parser extracts the entity “suggested time slot”, which is not extracted by the constituency parser.

**Table 10:** Example requirements for which one parser extracted an entity missed by the other, and vice versa.

#	Requirement	Entities Extracted by Constituency Parser	Entities Extracted by Dependency Parser
1	The system shall allow the admin to view the profile of a doctor.	admin, profile of a doctor, profile, doctor	admin, profile, doctor
2	The system shall notify the patient to confirm the newly suggested time slot.	newly suggested time slot, patient, time slot, time, slot	newly suggested time slot, suggested time slot, time slot, patient, time, slot

### Performance and error analysis by noun phrase categories

This section deals with performance evaluation and error analysis of noun phrase extraction by constituency parser, dependency parser, and hybrid approach with respect to noun phrase categories. The true positives (TP), false positives (FP), false negatives (FN), precision, recall, and F1-score are presented in Table 11.

**Table 11:** Performance values for occurrence-level entities extracted by all parsing techniques.

Noun Phrase Category	Technique	TP	FP	FN	Precision	Recall	F1-Score
Simple NP	Constituency Parser	3410	47	89	**98.64**	97.46	**98.04**
	Dependency Parser	3434	98	65	97.23	98.14	97.68
	Hybrid	3441	103	58	97.09	**98.34**	97.71
Complex NP	Constituency Parser	1181	19	50	98.42	95.94	97.16
	Dependency Parser	1045	16	186	**98.49**	84.89	91.19
	Hybrid	1204	24	27	98.05	**97.81**	**97.93**
Participial NP	Constituency Parser	145	2	29	**98.64**	83.34	90.34
	Dependency Parser	148	6	26	96.10	85.06	90.24
	Hybrid	165	11	9	93.75	**94.83**	**94.29**
Possessive NP	Constituency Parser	41	5	24	89.13	63.08	73.87
	Dependency Parser	47	1	18	97.92	72.31	83.19
	Hybrid	48	1	17	**97.96**	**73.85**	**84.21**
Participial-Possessive NP	Constituency Parser	0	0	2	0	0	0
	Dependency Parser	2	0	0	100	100	100
	Hybrid	2	0	0	100	100	100
Participial-Infinitival NP	Constituency Parser	1	0	0	100	100	100
Prepositional-Possessive NP	Constituency Parser	1	0	10	100	9.09	16.67
Participial-Prepositional NP	Constituency Parser	59	6	41	90.77	59.00	71.52
Prepositional Phrase-Based NP	Constituency Parser	179	4	123	97.81	59.27	73.81
Multiple Participial Chains	Constituency Parser	7	3	6	70.00	53.85	60.87
Infinitival NP	Constituency Parser	3	1	2	75.00	60.00	66.67
Prepositional-Infinitival NP	Constituency Parser	4	0	1	100	80.00	88.88
Complex NP with a Relative Clause	Constituency Parser	14	9	1	60.87	93.34	73.68
Participial-Prepositional-Infinitival NP	Constituency Parser	5	5	0	50	100	66.67

For the simple NP category, the constituency parser achieves the highest precision and F1-score due to its lower FP but reduced recall due to missing phrases. The dependency parser has higher FP and hence reduced precision, which can be attributed to the extraction of additional tokens (example: WDT, WP) which are not valid nouns or noun phrases. The hybrid approach achieves the highest recall due to the combined effect of the constituency parser and dependency parser, resulting in reduced FN. The additional noisy tokens inherited from the dependency parser slightly increase FP and hence reduce its precision and F1-score.

For the complex NP category, the constituency parser achieves the highest precision, and the dependency parser attains comparable precision. The dependency parser exhibits a considerably higher FN, which can be attributed to missing valid noun phrase extractions embedded within larger compound noun structures, resulting in reduced recall and F1-score. The hybrid approach recovers many of the noun phrases missed by the individual methods, particularly those missed by the dependency parser, leading to improved recall and the highest F1-score for this category.

The constituency parser attains the highest precision due to the reduced number of incorrect noun phrase extractions for the participial NP category. The dependency parser achieves performance comparable to that of the constituency parser. The highest recall and F1-score are achieved by the hybrid approach. It recovers more valid noun phrases, which results in a lower FN. However, the precision is slightly compromised due to the increased FP.

The dependency parser and the hybrid approach achieve high precision for the possessive NP category due to their low FP. However, considerably higher FN suggests that some valid possessive noun phrases remain undetected. The hybrid approach has the lowest FN and it achieves the highest recall and F1-score, indicating improved coverage of possessive noun phrases. In contrast, the constituency parser shows comparatively lower precision, recall, and F1-score due to higher FP and FN.

In case of the participial-possessive NP category, the constituency parser fails to extract any noun phrase while the dependency parser extracts both phrases. The hybrid approach also captures both phrases due to the combined effect of both parsers. For the participial–infinitival NP category, the constituency parser achieves perfect performance with one TP and zero FP and FN. The dependency parser does not extract this phrase. However, the small number of extracted phrases in these two categories limits the significance of these results.

In case of prepositional-possessive NP extractions, the constituency parser extracts only a limited number of phrases, resulting in high precision and reduced recall. Whereas in the participial-prepositional-possessive NP category, none of the approaches capture the annotated instances (7). The results indicate that the possessive noun phrases, involving complex structural complexity, are difficult to extract for parsers, leading to higher FN in these categories.

In the participial - prepositional NP, prepositional phrase-based NP, multiple participial chains, infinitival NP, and prepositional - infinitival NP categories, the constituency parser achieves moderate to high coverage, but the FN is relatively higher. It indicates that it is difficult to capture full phrase boundaries in longer or embedded structures by the constituency parser. The precision is high, but recall and F1-score are significantly reduced due to incomplete extractions.

The low FN and high FP are evident in complex noun phrases with a relative clause and participial-prepositional-infinitival NP categories. The constituency parser successfully identifies the presence of noun phrases but also extracts noun phrases that do not exactly follow the intended noun phrase boundary identified manually. These mismatches are mainly associated with complex and embedded structures. The extractions of the parser are correct, but these do not match with the manual phrasal boundaries due to their complexity.

For the prepositional-possessive NP, participial-prepositional-possessive NP, participial-prepositional NP, prepositional phrase-based NP, multiple participial chains, infinitival NP, prepositional-infinitival NP, complex NP with relative clause, and participial-prepositional-infinitival NP categories, the dependency parser extracts limited or no noun phrases, resulting in a high value of FN. The hybrid approach obtains limited additional benefit from the dependency parser in these categories, and its performance is largely determined by the constituency parser.

### Entity extraction performance project-wise

In this section, the precision, recall, and F1-score for each project by using a constituency parser, dependency parser, and the hybrid approach are presented and analysed.

A comparison of the precision for all nine projects by constituency parser, dependency parser, and hybrid approach is shown in Fig 7. The constituency parser gives the highest precision for P1, P2, P3, P4, P6, and P8. Whereas for projects P5, P7, and P9, the dependency parser gives the higher precision.

**Fig 7:**
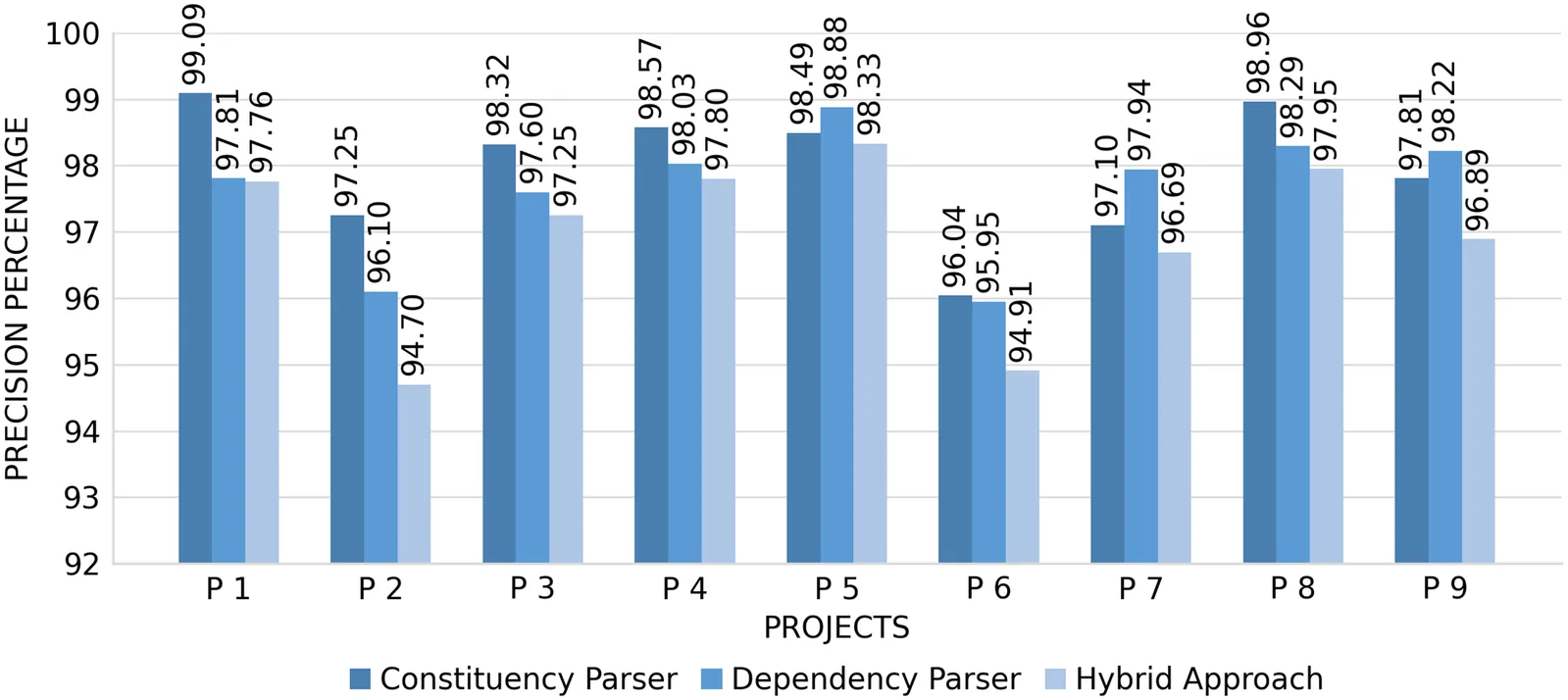
Precision results of all parsing techniques project-wise.

The precision of project-wise unique entities presented in Table 12 indicates that for projects P2, P4, P5, P6, P7, and P9, the dependency parser gives better performance, while for P1, P3, and P8, the constituency parser has a higher precision. Although TP for these projects is higher with the constituency parser, but the greater FP indicates that the constituency parser has extracted some long and structurally complex phrases that are not annotated manually in these projects. The precision of all the approaches is very close to each other, but it is evident that the precision of the hybrid approach is consistently lower due to the increased FP from the combined effect of the constituency and dependency parser.

**Table 12:** Performance values for project-wise unique entities extracted by all parsing techniques.

Project	Technique	TP	FP	FN	Precision	Recall	F1-Score
P1	Constituency Parser	212	8	41	**96.36**	83.79	89.64
	Dependency Parser	181	8	72	95.77	71.54	81.90
	Hybrid	218	10	35	95.61	**86.17**	**90.64**
P2	Constituency Parser	373	19	57	95.15	86.74	90.75
	Dependency Parser	315	13	115	**96.04**	73.26	83.12
	Hybrid	389	27	41	93.51	**90.47**	**91.96**
P3	Constituency Parser	167	4	23	**97.66**	87.89	92.52
	Dependency Parser	147	4	43	97.35	77.37	86.22
	Hybrid	169	6	21	96.57	**88.95**	**92.60**
P4	Constituency Parser	285	8	49	97.27	85.33	90.91
	Dependency Parser	254	6	80	**97.69**	76.05	85.52
	Hybrid	295	11	39	96.41	**88.32**	**92.19**
P5	Constituency Parser	260	6	27	97.74	90.59	94.03
	Dependency Parser	214	4	73	**98.17**	74.56	84.75
	Hybrid	261	7	26	97.39	**90.94**	**94.05**
P6	Constituency Parser	254	20	37	92.70	87.29	89.91
	Dependency Parser	221	15	70	**93.64**	75.95	83.87
	Hybrid	259	22	32	92.17	**89.00**	**90.56**
P7	Constituency Parser	89	4	15	95.70	85.58	90.36
	Dependency Parser	76	2	28	**97.44**	73.08	83.52
	Hybrid	91	5	13	94.79	**87.50**	**91.00**
P8	Constituency Parser	96	2	9	**97.96**	91.43	94.58
	Dependency Parser	84	2	21	97.67	80.00	87.96
	Hybrid	97	3	8	97.00	**92.38**	**94.63**
P9	Constituency Parser	117	4	12	96.69	90.70	93.60
	Dependency Parser	104	3	25	**97.20**	80.62	88.14
	Hybrid	124	6	5	95.38	**96.12**	**95.75**

The recall comparison for all nine projects based on the entity extraction by constituency parser, dependency parser, and hybrid approach is shown in Fig 8. For all projects, the hybrid approach gives the highest recall, whereas the dependency parser exhibits the lowest recall because it does not identify a higher number of noun phrases which are present in manual annotation.

**Fig 8:**
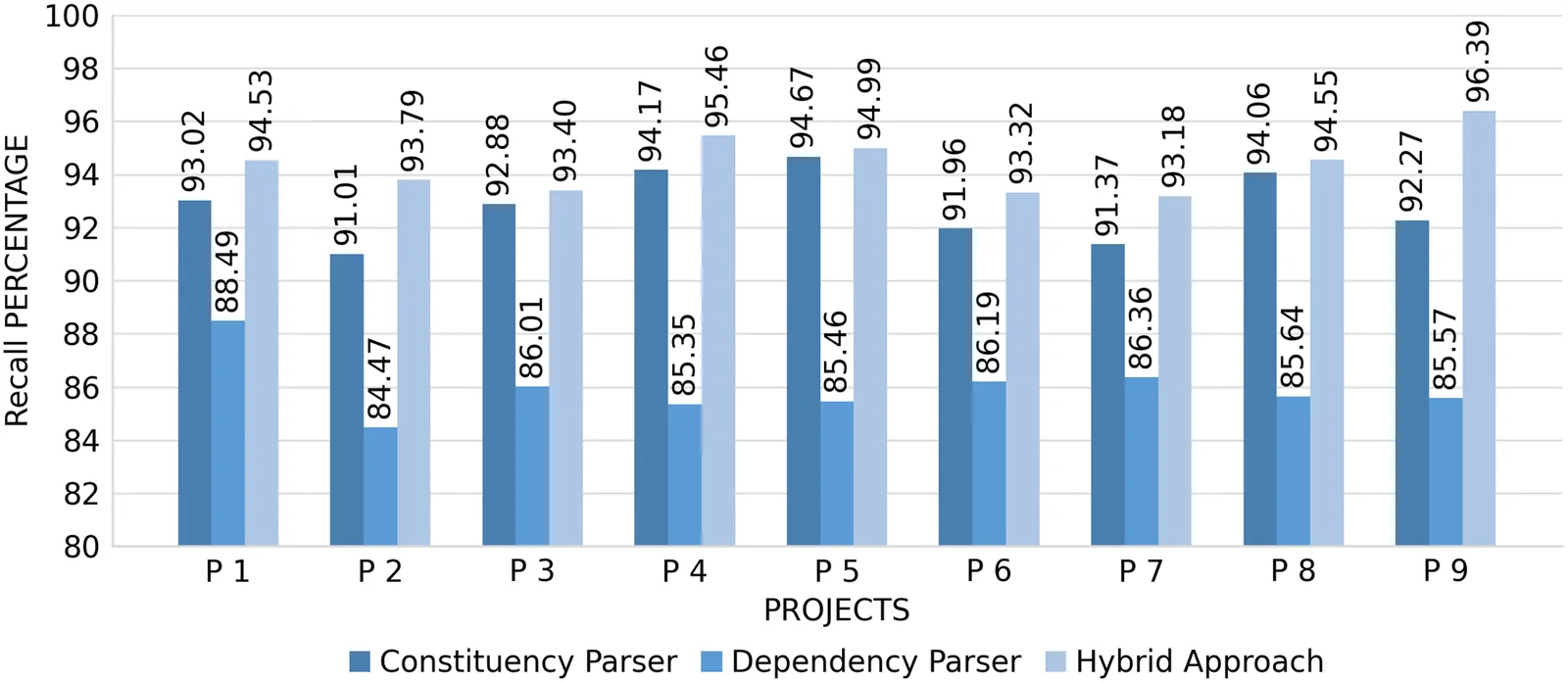
Recall of all parsing techniques, project-wise.

A similar trend for recall is observed for project-wise unique entities presented in Table 12. The dependency parser relies on head-dependent relations, and therefore, noun phrases having some postmodifiers are not identified by the dependency parser. As the constituency parser forms a hierarchical tree and supports recursive extractions, a wide range of recursive noun phrases are identified. A smaller number of compound nouns and deep recursive postmodifier-based noun phrases are missed by the constituency parser. Therefore, its recall is much higher than that of the dependency parser. The hybrid approach addresses these limitations by combining the outputs of both parsers. That’s why the hybrid approach gives the highest recall across all the projects.

The comparison of F1-score for all nine projects in terms of entity extraction is represented in Fig 9. The analysis of the F1-score indicates that the F1-score is lowest for the dependency parser for all nine projects. However, the results of the F1-score by the constituency parser and the hybrid approach are comparable. The F1-score by constituency parser is higher for P3 and P8, which indicates that the constituency parser is giving higher precision without a major recall loss. For all the remaining projects, the hybrid approach gives the highest F1-score reflecting a better precision-recall balance.

**Fig 9:**
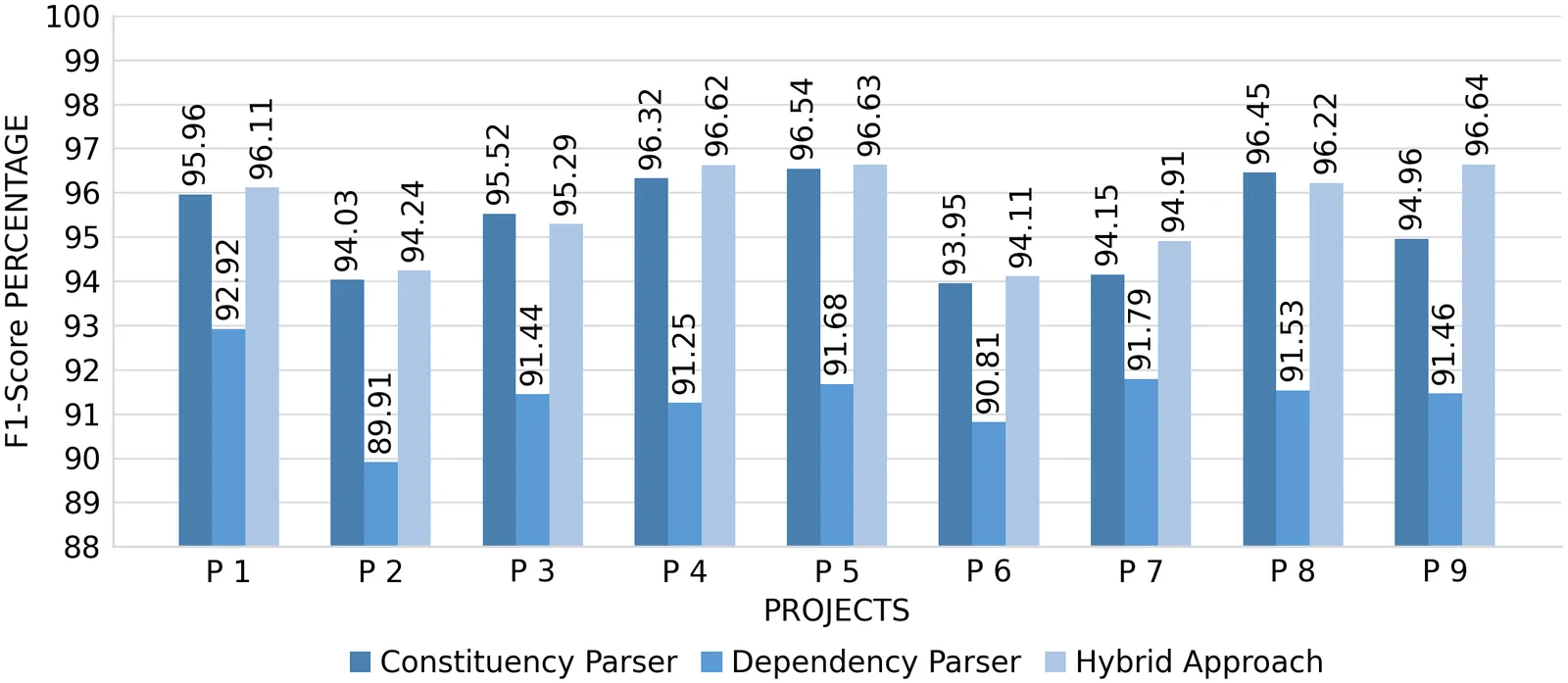
F1-score of all parsing techniques project-wise.

As indicated in Table 12, the hybrid approach achieves the highest F1-score for unique entity extraction in all nine projects, whereas it is lowest for the dependency parser. This is due to a substantial recall loss of project-wise unique entity extraction by the dependency parser. Although its precision is comparable to that of the constituency parser and the hybrid approach, the lower recall reduces the number of extracted entities, leading to a reduced F1-score.

To assess the variability in the performance of the constituency parser, dependency parser and hybrid approach across the nine software projects, the mean and standard deviation (SD) of the project-wise precision, recall, and F1-score are computed for both unique and overall entity extraction. For unique entity extraction, the hybrid approach achieves the highest mean F1-score (92.60 ± 1.85), followed by the constituency parser (91.81 ± 1.89) and the dependency parser (85.00 ± 2.15). For overall entity extraction across all nine projects, the hybrid approach achieves the highest mean F1-score (95.64 ± 1.03), followed closely by the constituency parser (95.32 ± 1.08), while the dependency parser obtains the lowest average F1-score (91.42 ± 0.80). The relatively small standard deviations indicate that the F1-scores of all three approaches are reasonably consistent across the evaluated projects. The results of mean and standard deviation for the project-wise precision, recall, and F1-score are presented in Table 13.

**Table 13:** Results of Mean and Standard Deviation for Project-wise Precision, Recall and F1-Score.

Technique	Mean ± SD for Unique Entities			Mean ± SD for Overall Entities		
	**Precision**	**Recall**	**F1-Score**	**Precision**	**Recall**	**F1-Score**
**Constituency Parser**	96.36 ± 1.67	87.70 ± 2.69	91.81 ± 1.89	97.96 ± 1.00	92.82 ± 1.29	95.32 ± 1.08
**Dependency Parser**	96.77 ± 1.41	75.83 ± 3.10	85.00 ± 2.15	97.65 ± 0.99	85.95 ± 1.10	91.42 ± 0.80
**Hybrid Approach**	95.43 ± 1.71	89.98 ± 2.96	92.60 ± 1.85	96.92 ± 1.31	94.40 ± 1.09	95.64 ± 1.03

To identify whether the performance improvements between parsing techniques are consistent across the nine projects for unique and overall entity extraction, the Wilcoxon signed-rank test [44] is performed for pairwise comparisons between the constituency, dependency, and hybrid approaches. Since the approaches are evaluated on the same projects, the resulting F1-scores constituted paired observations. The tests are performed using the SciPy statistical library in Python. The statistical analysis reveals different trends for the two evaluation settings.

For unique entity extraction, the hybrid approach achieves significantly higher project-wise F1-scores than both the constituency parser and the dependency parser (p = 0.00195 that is less than 0.05). The constituency parser significantly outperforms the dependency parser (p = 0.00195). So, it is evident that the hybrid approach consistently shows better performance for unique entity extraction across the all nine projects.

For overall entity extraction, the hybrid approach achieves higher F1-score than the constituency parser in seven of the nine projects. However, the project-wise improvement is not statistically significant (p = 0.102 that is greater than 0.05), suggesting that while the hybrid approach generally provides better performance, its performance improvement over the constituency parser is modest at the project level. However, both the hybrid approach and constituency parser achieve significantly higher F1-scores than the dependency parser (*p* = 0.00195).

Overall, the statistical analysis indicates that the complementary strengths of the two parsing techniques are more evident in unique entity extraction, where combining the outputs of both parsers leads to a statistically significant improvement over the individual parsing techniques.

### Combined entity extraction performance for all projects

In this section, the combined results for all nine projects are discussed. Table 14 shows the comparison of precision, recall, and F1-score of unique entities using the constituency parser, dependency parser, and hybrid approach. Fig 10 presents the corresponding comparison for all extracted entities across the three approaches.

**Table 14:** Performance values for unique entities of projects extracted by all parsing techniques.

Technique	TP	FP	FN	Precision	Recall	F1-Score
Constituency Parser	1414	60	261	95.93	84.42	89.81
Dependency	1165	36	510	**97.00**	69.55	81.02
Hybrid	1462	76	213	95.06	**87.28**	**91.01**

**Fig 10:**
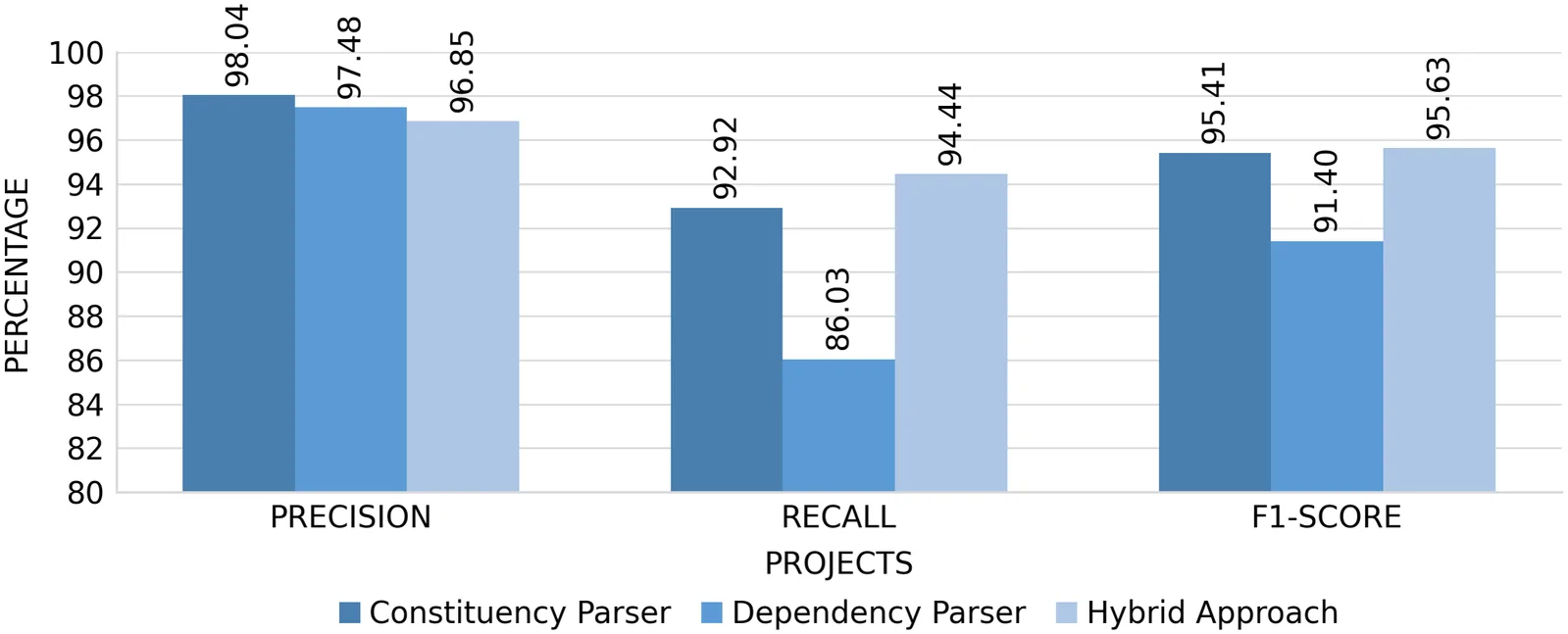
Combined precision, recall, and F1-score of projects for all parsing techniques.

The results in Table 14 indicate that for unique entity extraction, the dependency parser gives the highest precision, but there is a substantial compromise on recall and F1-score. This is also reflected in the higher FN and lower TP and FP of the dependency parser. It indicates that it extracts fewer entities compared to the constituency parser and the hybrid approach. From Fig 10 it is reflected that for total entities extraction, the constituency parser achieves the highest precision, which can be attributed to repeated correct extractions that substantially increase TP more than FP. The precision of the hybrid approach is very close to that of the constituency parser and the dependency parser.

The recall and F1-score are highest for the hybrid approach for both the total entities extraction and unique entities extraction, which indicates the balanced performance in terms of accuracy and coverage.

For the entity extraction task, recall is more important, as a low recall indicates that many entities that should have been identified are not extracted. A low precision can be overcome by removing unnecessary entities from the list of extracted entities, but it is very difficult to address the low recall. Hence, the hybrid approach provides greater extraction coverage for entity extraction as compared to constituency parsing and dependency parsing individually.

The overall better performance of the hybrid approach may be attributed to the integration of constituency-based phrase structures and dependency-based grammatical relations, which contributes additional extraction capability. It enables the extraction of base noun phrases, noun phrases with premodifiers and postmodifiers, compound nouns, and individual noun tokens. Furthermore, the hybrid approach captures noun phrase variations without relying on stop word removal, restructuring, or lemmatization. These results demonstrate the benefit of combining complementary syntactic representations for broader entity coverage, while the comparison with the pretrained neural approach highlights the trade-off between learned contextual representations and explicit, traceable syntactic extraction.

## Threats to validity

### External validity

In this section, possible threats to validity, like whether the proposed approach can be generalized, are discussed. First, the dataset comprises requirement statements only, so it may produce different results for a dataset of a different nature, such as application reviews, application descriptions, and code documentation. Second, in the preprocessing step, the requirement statements are decomposed from compound statements to simple statements, which makes it suitable for noun phrase extraction using parsers. It may limit its applicability to more complex real-world requirement specifications. Third, the data used in this research is domain-specific. The results may differ for a dataset from a different domain.

Despite these limitations, the proposed approach is primarily based on parsing techniques and does not rely on the domain-specific training of a parser. It depends on the grammatical structure of the sentences and noun phrase patterns present in the sentences.

A variation in results is observed while evaluating the approach on the requirements from different project categories having different phrase structures indicating that syntactic complexity can influence extraction performance. In addition, the baseline and proposed approaches are evaluated on different datasets, which rely on syntactic parsing for noun phrase extraction. This indicates that the parsing-based approaches may have the potential to be adapted across datasets from different domains. However, additional experiments are still required on datasets from other domains to validate the generalizability of the proposed approach.

### Internal validity

These are the potential threats related to errors and biases in experiments. The entire pre-processing of data and entity extraction for creating the gold standard relies on human judgement by three annotators with expertise in requirements engineering, linguistics, and the NLP domain. Therefore, there is a chance of errors in manual entity annotation serving as the gold standard. Using empirically supported rules and applying them carefully helps reduce this threat. Additionally, applying Cohen’s Kappa statistics provides an analysis of the agreement between annotators and helps minimize errors caused by human judgement.

## Conclusion

The study highlights the strengths and weaknesses of three automated parsing approaches in detecting entities from a natural language requirements dataset of the healthcare domain. These parsing approaches include constituency parsing, dependency parsing, and a hybrid approach. The dependency parsing that is based on the transition-based mechanism identifies base noun phrases, compound nouns, individual nouns, and noun phrases with premodifiers by analyzing grammatical structures based on head-modifier relationships, making it suitable for short and rule-based noun phrases. In contrast, constituency parsing, which is a transformer-based approach, excels at extracting long noun phrases, including base noun phrases, noun phrases with premodifiers and postmodifiers, compound nouns, and individual nouns. The precision of constituency parsing is highest among all the approaches, but it is comparable to the hybrid approach.

The hybrid approach achieves the highest recall and F1-score in the entity extraction task on the evaluated dataset by addressing sentence fragmentation and maintaining the phrase boundary integrity. With the highest recall and F1-score and a comparable precision to that of the constituency parser, the hybrid approach provides a balanced solution for the entity extraction task within the scope of this dataset. Moreover, the improved performance of the NP method on the healthcare requirements dataset provided a basis for extending the constituency-based extraction procedure to capture a broader range of noun phrase spans. The pretrained neural-based approach provides contextual representations without manually defined extraction rules, whereas the parser-based approach provides explicit and traceable extraction rules without requiring task-specific model fine-tuning.

Our research can be extended in several directions. The approach may be evaluated to extract entities from the requirement dataset beyond the healthcare domain to assess its applicability to other domains. Moreover, the applicability of the approach can be examined on more diverse and less structured text sources, such as software descriptions, application reviews, and code documentation. The entities extracted by using the hybrid approach may be used for a downstream task, such as identifying coreferent entities to resolve entity ambiguities and reduce inconsistencies in requirement statements. This can help unify different terms used to represent the same entities to maintain consistency.
